# Editorial: CRISPR in *Nucleic Acids Research*

**DOI:** 10.1093/nar/gkw451

**Published:** 2016-06-10

**Authors:** Barry L. Stoddard, Keith Fox

**Affiliations:** 1 Division of Basic Sciences, Fred Hutchinson Cancer Research Center, 1100 Fairview Avenue N. A3-025, Seattle, WA 98109, USA; 2 Centre for Biological Sciences, Life Sciences Building 85, University of Southampton, Southampton SO17 1BJ, UK

In biology, like most scientific disciplines, revolutionary advances often arrive quietly, often in the form of phenomenological observations followed by initial mechanistic analyses. In many if not most cases, the importance of those studies is recognized only in retrospect. One of the most well-cited examples of a progression from curiosity-driven research, conducted by a small handful of individuals, to an epochal change in the scientific landscape can be found in the original descriptions of bacterial phage restriction and the subsequent isolation and characterization of the first restriction endonucleases (reviewed in (1)). Those breakthroughs are now credited with a critical role in the establishment of modern molecular biology and the formation of the biotechnology industry. At the time of their publication, however, the manuscripts describing those observations (eventually leading to multiple Nobel Prizes) appeared in what many researchers might describe as ‘specialized’ journals describing bacteriological and molecular research.

Fortunately, the scientific research community has a long memory, enabled by its clearly archived record of achievement, and most studies eventually garner the accolades and/or notoriety that they ultimately deserve. The seemingly esoteric and specialized genetic studies of host-controlled variation in phage infectivity that were described in the early 1950s (2,3) are now recognized as the intellectual gateway into a scientific and technological revolution that continues to play out almost 60 years later. Since its inception, the journal *Nucleic Acids Research* has taken a keen interest in (and served as a major publication venue for) studies of restriction endonuclease research and applications. A special online collection of historical reviews and perspectives of the history of restriction endonuclease research, and of recently published research articles on the subject in *NAR*, is available at http://www.oxfordjournals.org/our_journals/nar/restriction_enzymes_collection.html.

Now a new revolution, in the form of the development and application of the CRISPR/Cas9 gene targeting system, has swept through the academic and commercial biotech communities at a pace that can reasonably be described as breathtaking (4,5). Since the first description of how the CRISPR/Cas9 molecular complex can be organized and used for targeted gene modification (6–8), the number of studies citing ‘CRISPR + Cas9’, as indexed in PubMed, has exploded from three papers in 2012 to a projection of over 2000 publications in 2016. During that time period, the CRISPR/Cas9 platform has been used for targeted gene modification in almost all recognized eukaryotic model systems. It has also been employed for targeted regulation of gene expression, and more recently has been modified to create approaches for site-specific epigenetic modification (9).

The initial descriptions of bacterial CRISPR genetic loci, and of the presence of exogenous phage-derived sequences within those loci (eventually leading to hypotheses that CRISPR elements represent some form of acquired bacterial immunological memory) are found in a series of publications spanning a 10-year period from the early 1990s through the mid-2000s. Those papers, which in hindsight represent a collective breakthrough, led directly to the current era of gene targeting and genome engineering, and were largely published in journals focused on molecular evolution, microbiology and bioinformatics. That work culminated in publications from three laboratories in 2005 (10–12) that are all now recognized as seminal contributions to the field of targeted gene modification and genetic engineering. Each of those manuscript were initially submitted to several ‘higher impact’ journals (including, unfortunately, *Nucleic Acids Research*), but returned to the authors after editorial evaluation. However (as was the case for restriction enzymes) the attention that those studies deserve is now being realized.

Since 2005, additional studies from a diverse group of laboratories (spanning microbiologists, enzymologists, bioinformaticians and many additional disciplines) have produced a rapidly expanding body of literature that has collectively described the biological purpose, molecular and genetic composition, mechanisms of action and the ever-more complex ways to draw phylogenetic divisions and classification schemes for bacterial CRISPR systems (13). One particularly critical study, published in *Nucleic Acids Research* in 2011, demonstrated that the transplantation of an entire type II CRISPR locus from *Streptococcus thermophilus* into the distantly related *Escherichia coli* resulted in the transfer of targeted resistance against exogenous phage- and plasmid-derived foreign DNA (14). That same experiment also demonstrated that the Cas9 protein, and its associated HNH- and RuvC-like nuclease domains, are necessary and sufficient for CRISPR activity.

Since the publication of that study, *Nucleic Acids Research* has published over 70 articles on CRISPR biology, evenly distributed between studies that continue to unravel details of the formation of CRISPR loci and their subsequent mechanisms of surveillance and action, and studies that describe the application of CRISPR for targeted gene modification and genome engineering. This collection of publications is now organized at http://www.oxfordjournals.org/our_journals/nar/crispr_cas_collection.html. Included in this special online collection are mechanistic studies of the acquisition of spacer elements in CRISPR loci, characterization of individual protein and RNA components from many different classes of CRISPR systems, descriptions of CRISPR action across a wide range of bacterial hosts and demonstrations of a wide variety of applications of CRISPR gene targeting systems for many different purposes. Some of the most recent CRISPR publications at NAR include detailed biophysical analyses of the kinetics of CRISPR/Cas9 assembly (15) and one of the first reports of the development of a CRISPR/Cas9 based platform for targeted DNA methylation (9).

Moving forward, the field of CRISPR biology and applications clearly remains fertile ground for continued study. Many details of the mechanisms employed by CRISPR systems (which currently encompass at least two classes, five types and 16 subtypes (16)), and particularly those of the more complex multi-protein systems, remain to be elucidated. Variants of the prototypical Cas9 nuclease, including those with substantially different domain sizes, unique patterns of structural organization and distinct reaction mechanisms yielding differing DNA product ends, offer the possibility of unique properties and uses for biotechnology (17). *Nucleic Acids Research* looks forward to continuing to receive and publish such studies, and to the expansion of our online collection of CRISPR studies as the field continues to grow and mature.
