# Editorial: CRISPR in *Nucleic Acids Research*

**DOI:** 10.1093/nar/gkw618

**Published:** 2016-07-05

**Authors:** Barry L. Stoddard, Keith Fox

**Affiliations:** 1 Division of Basic Sciences, Fred Hutchinson Cancer Research Center, 1100 Fairview Avenue N. A3-025, Seattle, WA 98109, USA; 2 Centre for Biological Sciences, Life Sciences Building 85, University of Southampton, Southampton SO17 1BJ, UK

*Nucl. Acids Res*. (20 June 2016) 44 (11): 4989–4990. doi: 10.1093/nar/gkw451

The Editors inadvertently omitted reference 18, which should have been cited on page 4989 in the following sentence: “Since the first descriptions of how the CRISPR/Cas9 molecular complex can be organized and used for targeted gene modification (6–8,**18**), the number of studies citing ‘CRISPR + Cas9’, as indexed in PubMed, has exploded from **four** papers in 2012 to a projection of over 2000 publications in 2016.”
